# The association between leisure-time physical activity and blood pressure changes from adolescence to young adulthood: Tehran Lipid and Glucose Study

**DOI:** 10.1038/s41598-023-48253-8

**Published:** 2023-11-28

**Authors:** Reza Yari-Boroujeni, Mohammad-Farid Farjad, Keyvan Olazadeh, Leila Cheraghi, Parnian Parvin, Fereidoun Azizi, Parisa Amiri

**Affiliations:** 1 Research Center for Social Determinants of Health, Research Institute for Endocrine Sciences, Shahid Beheshti University of Medical Sciences, P.O.Box: 19395-4763, Tehran, Iran; 2 Department of Biostatistics, School of Allied Medical Sciences, Shahid Beheshti University of Medical Sciences, Tehran, Iran; 3 Department of Epidemiology and Biostatistics, Research Institute for Endocrine Sciences, Shahid Beheshti University of Medical Sciences, Tehran, Iran; 4 Research Institute for Endocrine Sciences, Endocrine Research Center, Shahid Beheshti University of Medical Sciences, Tehran, Iran

**Keywords:** Cardiology, Endocrinology, Medical research, Risk factors

## Abstract

The effectiveness of long-term leisure time physical activity (LTPA) on blood pressure (BP) changes is still under debate. Since adolescence lifestyle behaviors shape the adulthood health profile, this study aimed to investigate the sex-specific impact of LTPA on BP changes from adolescence to young adulthood. This longitudinal study uses the data of 1412 adolescents (52% females) aged 12–18 years through a median follow-up of 12.2 years in the Tehran Lipid and Glucose Study (TLGS) framework. LTPA was calculated using the reliable and valid Iranian version of the modified activity scale (MAQ), and BP was measured at least twice by trained physicians. The linear mixed model was used to examine the study variables, considering individual and intrapersonal differences during the study. The majority of participants consistently demonstrated insufficient LTPA throughout the follow-up assessments, ranging from 54.7 to 67.1% for males and 77.7–83.4% for females. Despite a declining trend in LTPA (β = − 2.77 for males and β = − 1.43 for females), an increasing trend was noticeable in SBP, DBP, and BMI (β = 1.38, β = 1.81, β = 0.97 for males, and β = 0.10, β = 0.20, β = 0.97 for females, respectively). The unadjusted model revealed a significant trend in all variables for both sexes, except for female BP (P = 0.45 for SBP and P = 0.83 for DBP). Using the adjusted model, no significant association was observed between LTPA and changes in BP over time in both sexes. Our study indicates no association between LTPA and BP changes from adolescence to young adulthood. Insufficient LTPA levels, particularly among Iranian females, are likely the primary factor. Further research is crucial to identify appropriate LTPA levels to promote cardiovascular health and implement targeted interventions to achieve optimal LTPA levels in the Iranian population.

## Introduction

Hypertension (HTN) is an important yet preventable risk factor for chronic diseases. The results of various studies have shown an upward trend in the worldwide prevalence of HTN during the last decade^1,2^. A recent report showed that 25% of Iranian adults suffer from HTN, which is almost similar to the global, and also the Middle East region prevalence of this disorder^3,4^. Strong evidence suggests that HTN in adulthood can be traced back to early adolescence^5,6^. Evidence shows that 10.3% of Iranian boys and 9.1% of girls (8.9% in total) suffer from HTN during childhood and adolescence which is almost equal to worldwide prevalence (9.67%), and less than Arab countries (12.6%)^7–9^. Further national investigation on adolescents confirm high constant trends of pre-HTN and HTN among Iranian boys and girls which changed from 14.23 to 29.18% and from 16.79 to 27.66% during a three-year follow-up, respectively, regardless of their residential areas^10^.

Age, sex, race, family history of high blood pressure, and unhealthy lifestyle, including excessive salt consumption, alcohol, and smoking are the most important underlying factors of HTN^10,11^. Meanwhile, the impact of physical activity (PA) on HTN as a modifiable behavior is still questioned. Some studies attribute 5–13% of HTN to inadequate PA^12^ and suggest adequate daily PA as an effective “polypill” for hypertension control^13^. However, in some other studies, no significant relationship between PA and blood pressure (BP) has been reported, and even its negative effects on BP status have been emphasized^14–17^. Regarding the long-term effects of PA on BP, although the available evidence favors the stronger effects of PA on some cardiovascular risk factors, including obesity and diabetes, the certainty of similar findings on BP is still under debate and requires further research^8^.

Longitudinal studies show that 81% of adolescents do not meet the recommended amount of activity, and they lose about a quarter of the amount of PA, which is equivalent to 1.01 MET per day during the transition from adolescence to adulthood^18,19^. As a country that experienced a deep lifestyle transition during the last decades, a medium to high insufficient PA has been observed among different Iranian communities, with a prevalence of 39.1% in the general population. This statistic is more than worldwide (27.5%) and also countries in central Asia, the Middle East, and North Africa (32.8%)^20,21^. According to available studies, about a quarter of Iranian children and adolescents are physically inactive^22^, which has an upward trend in both genders residing in urban and rural areas^11^. In the meantime, considering the sociocultural obstacles for women to be physically active in public places and their limited access to private gyms and sports complexes, Iran has one of the most significant sex differences in the lack of sufficient PA, with a score of 69.1% in women and 45.3% in men^23^.

Considering the relationship between the adolescents’ lifestyles, including PA, with their cardiometabolic health^24^ and also the high probability of stabilizing these behavioral patterns throughout life, the premise of the relationship between simultaneous trends of PA and BP from adolescence to later life seems logical. To the best of our knowledge, few studies and mostly in developed countries have been conducted to investigate the association of PA and BP only in adolescence^16,25,26^ or adulthood^27,28^, but the transition from adolescence to adulthood is still unclear, which necessitates the need to complement the literature. As one of the first efforts, the present study aimed to investigate the long-term sex-specific effects of PA changes on changes of BP from adolescence to young adulthood in an Iranian population in the framework of the TLGS as the oldest cardio-metabolic cohort in the Middle East.

## Methods

### Study design and population

The Tehran Lipid and Glucose Study (TLGS), a large-scale population-based cohort study with long-term follow-up, was conducted to identify risk factors associated with non-communicable diseases among a representative sample of the urban population in Tehran, the capital of Iran. Tehran Lipid and Glucose Study (TLGS) has been conducted since 1999 with a cross-sectional baseline assessment of risk factors and the prevalence of non-communicable diseases, which has continued to investigate the changes in the form of 3-year follow-up periods. More detailed information about the TLGS protocol, design, and data collection are discussed in other studies^29,30^.

Participants were selected from three healthcare centers in the 13th district of Tehran using a random cluster sampling method. Population stability, age distribution similar to the main population, socio-economic status, and access to complete family-documented data as a sample of people living in Tehran were among the reasons for choosing the mentioned district. From 7151 families, 15,005 individuals (age ≥ 3 years) participated in the TLGS baseline measurement after completing consent forms.

For the present study, data from 1567 adolescents (12–18 years) who participated in the second phase of the TLGS (2002–2004) has been considered. After excluding those with missing data on physical activity in all follow-ups (n = 155), 1412 participants remained for the final analysis. These participants were followed for a median of 12.2 years during four follow-up examinations.

### Measurements and definitions

#### Socio-demographic information

At baseline and in each follow-up evaluation, valid and reliable questionnaires for gathering socio-demographic data and PA were used by trained interviewers to obtain information from participants. Education at baseline was categorized based on the years of study as illiterate/primary (0–6 years) and secondary (6–12 years). The higher category of education was considered for adults who had academic education. Regarding marital status, participants were divided into married and single. Participants were categorized as employed and unemployed based on whether they had a job or not.

#### Leisure-time physical activity (LTPA)

A continuous dependent variable from adolescence to young adulthood was considered to evaluate the quantity and quality of an individual’s PA during leisure time (based on energy consumed as an indicator). Using the valid Iranian version of the Modifiable Activity Questionnaire (MAQ) for adolescents aged 12–18^31^ and adults^32^, which evaluates the amount of time spent in 15 popular Iranian leisure activities, the total weekly hours allocated to each activity (time spent in LTPA) was calculated. By multiplying the obtained values in metabolic equivalents (METs), the amount of energy consumed in LTPA was obtained in the unit of MET.h/week. Metabolic equivalent task (MET) is a physiological measure used to express the intensity of a specific activity, regardless of its duration^33^. The value of this index, which describes the energy consumption, is defined based on the reference metabolic rate, which is set to 3.5 ml O2/kg min according to the convention. In many studies, MET is defined as the energy consumed based on kilocalories per body weight per unit of time (1 MET = 1 kcal/(Kg*h)).

The psychometric properties of the Persian version of the MAQ questionnaires for adolescents and adults have already been examined^31,32^. For both adolescent and adult versions of the translated questionnaires, the calculated intraclass correlation coefficients were 0.97 and 0.94, respectively, indicated a strong reliability for both instruments. Pearson correlation coefficients between the mean of two MAQs and the mean of four LTPA records (in each mid-season during a year) were 0.49 (P < 0.001) and 0.39 (P = 0.05) for adolescent and adult versions, respectively.

According to the 2020 World Health Organization guideline on PA and sedentary behavior^34^, it is recommended that children and adolescents engage in an average of at least one hour per day of moderate-to-vigorous intensity PA, while adults should aim for at least 2.5–5 h of moderate-intensity activity per week to achieve substantial health benefits. In the context of this guideline^34^, moderate-intensity physical activity refers to activities performed at an intensity ranging from 3 to less than 6 times that of rest (METs). In this study, participants were categorized based on their levels of LTPA. Adolescents who engaged in less than 30 MET-hour/week of LTPA were classified as having insufficient LTPA, and similarly, adults who participated in less than 16 MET-hour/week of LTPA were placed in the same group.

*Cigarette smoking.* During each follow-up measurement, smoking data were gathered through the utilization of standardized questionnaires. Smoking quantity (i.e., the number of cigarettes smoked per day) and smoking frequency (i.e., the number of days engaged in smoking within the past 30 days) were reported by adolescents (aged 18 or younger) and utilized to classify individuals into two groups: smokers and non-smokers. On the other hand, a question pertaining to current smoking status was answered by adults (aged over 18) with response options of “yes, every day,” “yes, sometimes,” or “no.” In cases where an affirmative response to the initial question was provided, an assessment was conducted regarding the number of cigarettes consumed per day (quantity) and the frequency of smoking over the preceding seven days (frequency).

#### Anthropometric data

A digital scale (Seca 707: range 0–150 kg with an accuracy of 100 g) and a tape meter stadiometer were used to measure weight and height with standard protocols. Participants took off their shoes and heavy clothing to measure weight and height accurately, and their heads, shoulders, buttocks, and heels stuck to the wall. Body mass index (BMI) was defined as the body mass divided by the square of the body height in the unit of kg/m^2^.

#### Blood pressure

For taking BP, participants rested for 15 min. BP was measured twice at least 30s intervals using a calibrated standard mercury sphygmomanometer appropriate to the participant’s arm. The mean of estimated BP was reported. The cuff was emptied at a speed of 2–3 mmHg/s after filling 30 mmHg over the pressure in which the radial pulse could not be palpated. The first and fifth Korotkoff sounds were defined as systolic and diastolic blood pressure (SBP and DBP), respectively. The same study personnel recorded all measurements to remove subjective errors.

#### Parental metabolic syndrome

Based on the joint interim statement^35^, the presence of any three of the following five risk factors was considered as the presence of metabolic syndrome in the parents: (1) waist circumference ≥ 90 cm for both sexes; (2) high-density lipoprotein cholesterol (HDL-C) < 50 mg/dl in female, < 40 in male, or receiving medications for reduced HDL-C; (3) elevated triglyceride (TG) levels ≥ 150 mg/dl or receiving medications for elevated TG; (4) elevated BP (≥ 130 mmHg SBP or ≥ 85 mmHg DBP) or receiving antihypertensive medications and (5) elevated FBG ≥ 100 mg/dl or receiving medications for elevated FBG.

### Statistical methods

Weighted predictive mean matching (WPMM) was chosen as the best method after comparing several methods with each other and regarding the nature of the data^36^. The five data sets were generated by the WPMM method. The linear mixed-effects model was fitted to each of these 5 data sets for boys and girls, and finally, the results were pooled in each sex separately. As an extension of simple linear models, the linear mixed model allows both fixed and random effects, preventing false positive associations due to population or related structure^37^. This model is valid because it uses individual and interpersonal differences over time. Also, an unstructured matrix was used to show the correlation in the linear mixed effects model. This model considered age, marital status, education, smoking, BMI,and parental Mets as adjusting factors. To intuitively interpret the trend of LTPA and, SBP, DBP over time, an individualized profile plot was drawn for a random sample of 300 participants (50% female).

All statistical analyses were performed using R-Studio software version 2022.07.1. Various packages were used for imputation, preliminary analyses, and fitting linear mixed effects models. The essential packages used in our study were the mice package for imputation and the lme4 package for fitting the linear mixed-effect model. Also, the ggplot2 package in R-Studio software was used to draw the graphs in this study.

### Ethics approval and consent to participate

This study was approved by the Ethical Committee of Research Institute for Endocrine Sciences and the National Research Council of the Islamic Republic of Iran (Ethic No: 121). Informed consent was obtained from all individual participants included in the study. All procedures were in accordance with the ethical standards of the institutional and/or national research committee and with the 1964 Helsinki declaration and its later amendments or comparable ethical standards.

## Results

Data on 1412 adolescents (48.0% males), aged 15.20 ± 1.94, who participated in the TLGS from 2002 to 2004 were recruited in the current analysis and followed for a median of 12.2 years. At baseline assessment, most adolescent males and females were single, unemployed, non-smoker, and had insufficient amount of LTPA. The mean LTPA and SBP were higher in males (P < 0.001), and the mean DBP was higher in females (P = 0.013) (Table 1).

**Table 1:** Baseline characteristics of participants in adolescence.

	Males (n = 678)	Females (n = 734)	P-value
Age	15.14 ± 1.94	15.51 ± 1.95	0.292
Education (%)			0.105
Illiterate/Primary	558 (82.3)	579 (78.9)	
Secondary	120 (17.7)	155 (21.1)	
Marital status (%)			**0.009**
Married	1 (0.1)	10 (1.4)	
Single	677 (99.9)	724 (98.6)	
Occupation (%)			** < 0.001**
Employed	35 (5.2)	5 (0.7)	
Unemployed	643 (94.8)	729 (99.3)	
Smoking (%)			** < 0.001**
Yes	121(17.8)	83(11.3)	
No	557(82.2)	651(88.7)	
BMI (kg/m^2^)	21.63 ± 4.54	21.58 ± 4.03	0.783
Maternal MetS (%)			0.433
Yes	288 (42.5)	327 (44.6)	
No	390 (57.5)	407 (55.4)	
Paternal MetS (%)			0.500
Yes	377 (55.6)	395 (53.8)	
No	301 (44.4)	339 (46.2)	
SBP (mmHg)	105.50 ± 11.85	100.44 ± 10.94	** < 0.001**
DBP (mmHg)	67.69 ± 9.35	68.92 ± 9.20	**0.013**
LTPA (MET-hour/week)	27.97 ± 32.54	14.06 ± 17.69	** < 0.001**
LTPA status (%)			<0.001
Insufficient	455 (67.1)	606 (82.6)	

Values are Mean (SD) for continuous variables and n (%) for categorical variables.

The p-value is for comparison between males and females in each characteristic.

BMI; body mass index, SBP; systolic blood pressure, DBP; diastolic blood pressure, LTPA; leisure time physical activity.

Significant values are in [bold].

**Figure 1:**
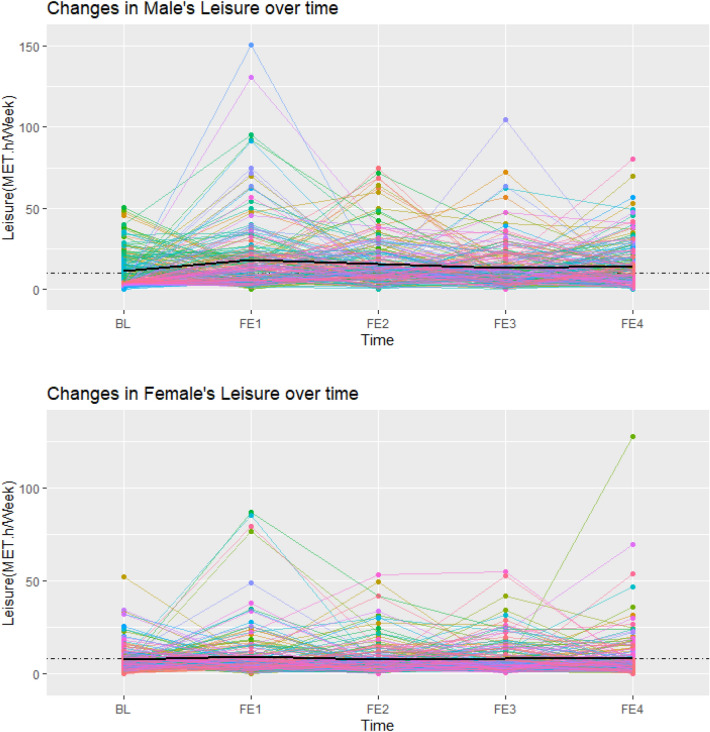
Individual profile plot for changes in leisure time physical activity in male and female participants from baseline to the last follow-up examination (Each colored line is assigned to one individual, and the black line represents the mean LTPA across all individuals).

**Figure 2:**
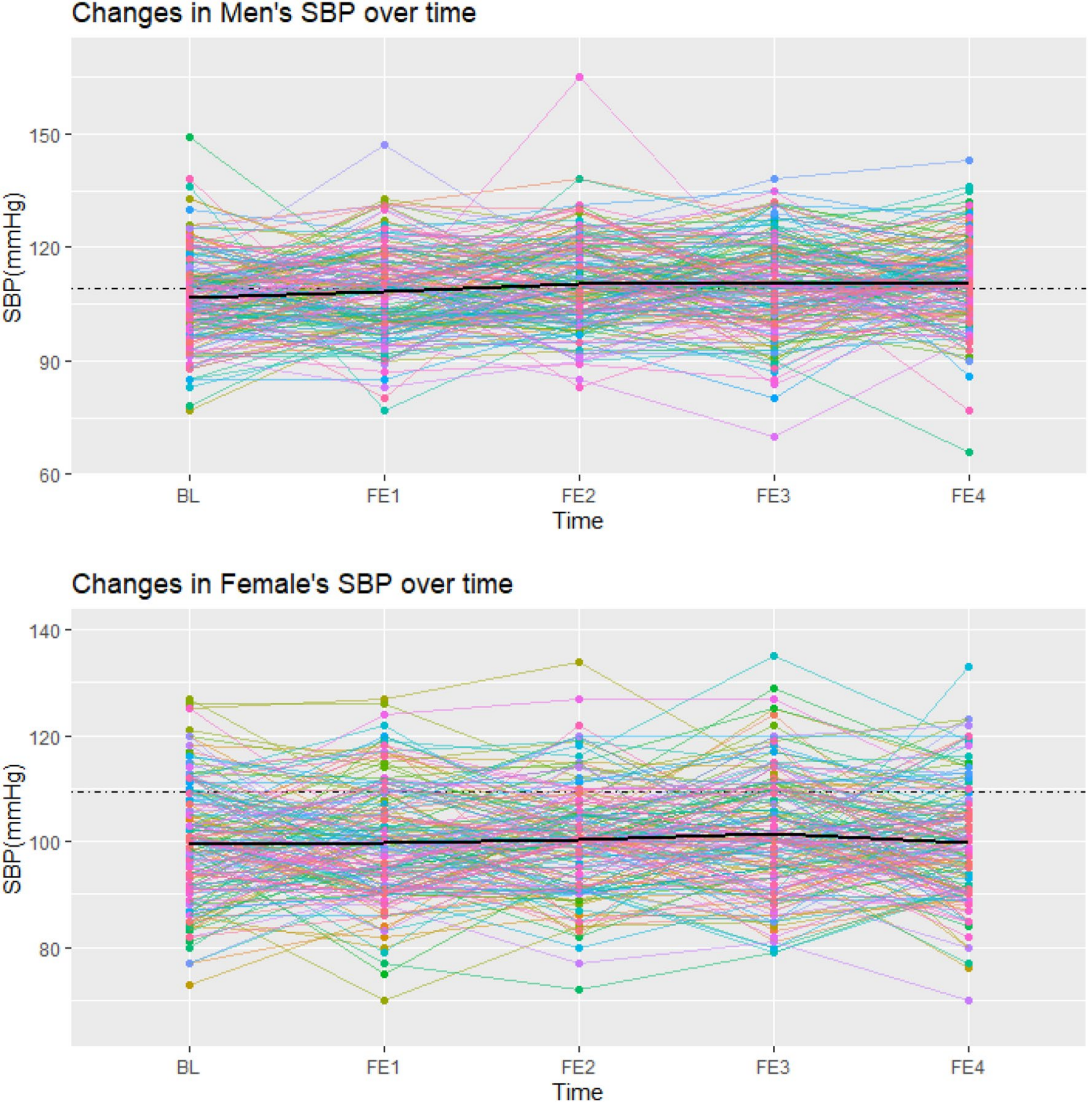
Individual profile plot for changes in SBP in male and female participants from baseline to the last follow-up examination (Each colored line is assigned to one individual, and the black line represents the mean SBP across all individuals).

**Figure 3:**
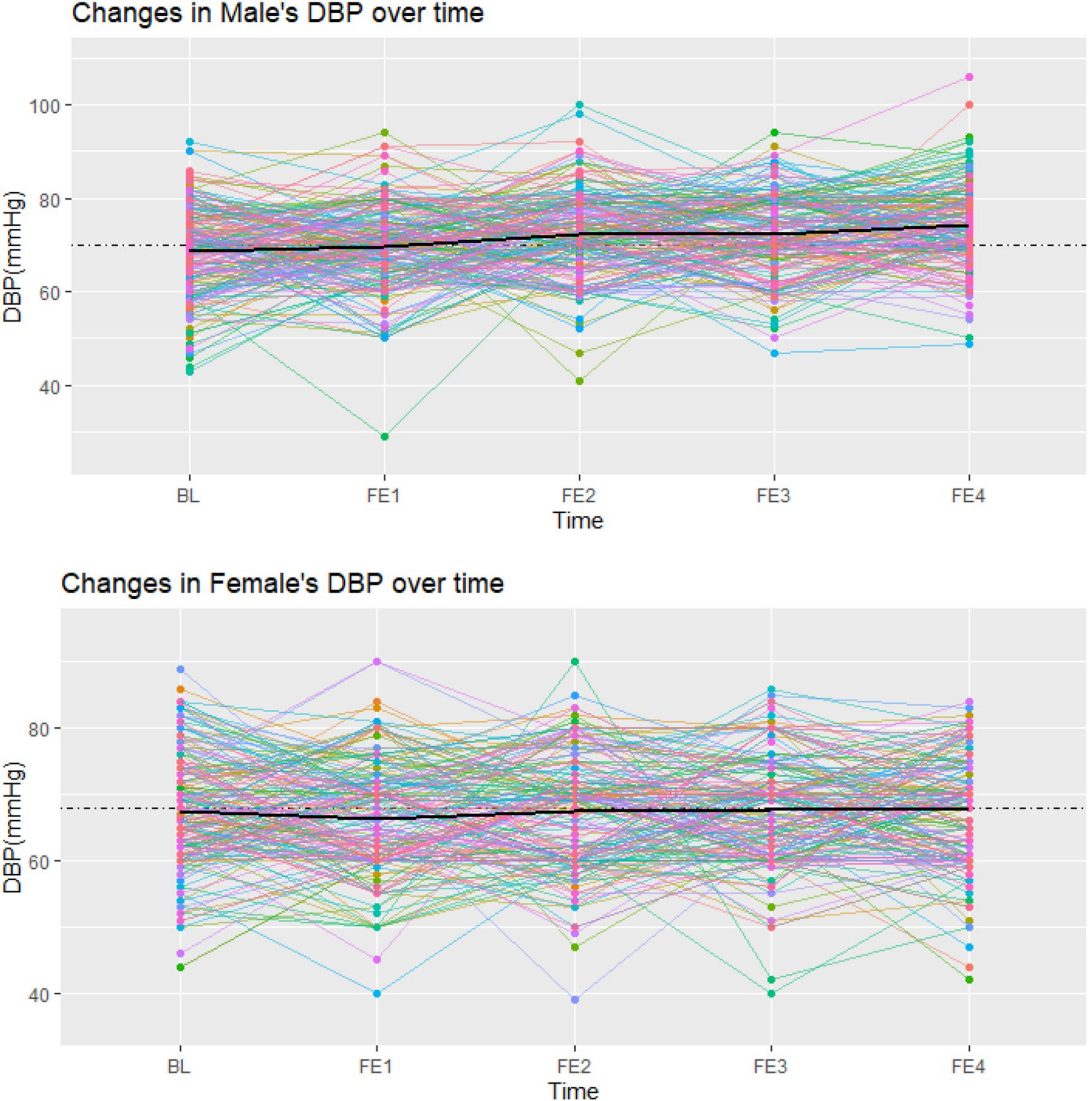
Individual profile plot for changes in DBP in male and female participants from baseline to the last follow-up examination(Each colored line is assigned to one individual, and the black line represents the mean DBP across all individuals).

Figures 1, 2, and 3 showed individual profile plots conducted on the specified random sample of participants (150 male and 150 female) regarding LTPA, SBP, and DBP. Intrapersonal changes during follow-up examinations indicate the possible variability in the slope and intercept of variables in both sexes. In most participants, the mentioned variables changed within limited ranges over time; while a few participants showed fluctuations in their SBP, DBP, and LTPA between some of the follow-up examinations, which was more dominant in the last profile. Most of these fluctuations in all variables were observed in males compared to females. In addition, the mean values of all study variables were higher in males compared to females (presented by black lines).

Table 2 represents the unadjusted mean values of age, SBP, DBP, LTPA and BMI, besides smoking and insufficient LTPA frequency through the four follow-up examinations in both sexes. Considering each follow-up examination, all the mentioned variables were significantly different between males and females except for age. In terms of changes in variables during the study assessments, increased trends were observed in SBP (β = 1.38 in males, and β = 0.10 in females), DBP (β = 1.81 in males, and β = 0.02 in females), and BMI (β = 0.97 in males, and β = 0.61 in females). LTPA demonstrated a decrease, as indicated by β values of − 2.77 for males and − 1.43 for females. In terms of insufficient LTPA frequency, males typically showed a declining pattern, whereas females did not exhibit a clear trend. As for smoking frequency, male participants displayed an increasing trend, while no specific pattern was observed among their female counterparts.The trend of these changes differed significantly in all variables for both sexes except for the female's BP (P = 0.45 for SBP and P = 0.83 for DBP).

**Table 2:** Study variables through four follow-up examinations in males and females.

	Male						Female					
	Follow-up examination 1	Follow-up examination 2	Follow-up examination 3	Follow-up examination 4	Trend		Follow-up examination 1	Follow-up examination 2	Follow-up examination 3	Follow-up examination 4	Trend	
					β	P-value					β	P-value
Age (Year)	18.14 ± 2.37	21.76 ± 2.48	25.06 ± 2.46	28.08 ± 2.42	3.27	< 0.001	18.33 ± 2.24	21.71 ± 2.34	25.03 ± 2.49	28.08 ± 2.46	3.23	< 0.001
Smoking (%)	118 (17.4)	158 (23.3)	185 (27.4)	207 (30.7)	–	–	46 (6.3)	32 (4.4)	38 (5.2)	50 (6.8)	–	–
BMI (kg/m^2^)	23.54 ± 4.9	24.72 ± 5.05	24.96 ± 4.88	25.78 ± 5.27	0.97	< 0.001	22.43 ± 4.08	23.36 ± 4.3	23.72 ± 4.43	23.96 ± 4.53	0.61	< 0.001
SBP (mmHg)	108.46 ± 11.38	111.20 ± 12.07	110.79 ± 11.85	111.2 ± 11.32	1.38	< 0.001	99.10 ± 10.82	100.30 ± 10.73	101.04 ± 11.31	99.99 ± 10.31	0.10	0.45
DBP (mmHg)	70.37 ± 8.89	73.26 ± 9.07	73.58 ± 8.50	75.13 ± 8.38	1.81	< 0.001	66.34 ± 8.81	67.81 ± 8.89	68.09 ± 8.68	68.14 ± 8.10	0.02	0.83
LTPA (MET-hour/week)	21.21 ± 23.75	18.31 ± 20.76	17.21 ± 18.15	16.12 ± 16.10	-2.77	< 0.001	11.05 ± 15.82	9.36 ± 11.19	8.53 ± 10.10	8.19 ± 10.27	-1.43	< 0.001
Insufficient LTPA (%)	445 (65.6)	407 (60.0)	371 (54.7)	389 (57.4)	–	–	612 (83.4)	570 (77.7)	586 (79.8)	576 (78.5)	–	–

All the mentioned variables were significantly different between males and females except for age in each follow-up examination (P < 0.001).

β represents the difference in the mean value of each variable over follow-up examinations.

Values are Mean (SD) for continuous variables.

BMI, body mass index; SBP, systolic blood pressure; DBP, diastolic blood pressure; LTPA, leisure time physical activity.

The adjusted linear mixed model (Table 3) revealed that, in both sexes, each MET-hour/week increase in LTPA did not result in significant changes in SBP (β = − 0.004, P = 0.595 and β = 0.006, P = 0.729, for males and females, respectively), and DBP (β = − 0.009, P = 0.269 and β = 0.027, P = 0.111 for males and females, respectively). Regarding influential factors within the adjusted model, increasing age was associated with rising in males BP (β = 0.425, P = 0.032 for SBP, and β = 0.426, P = 0.003 for DBP), and also females DBP (β = 0.199, P = 0.034). In male participants, compared to primary education, secondary education was linked to higher BP (β = 2.153, P = 0.004 and β = 1.563, P = 0.006 for SBP and DBP, respectively), while higher education was only correlated with higher SBP (β = 2.503, P = 0.020). In addition, employed participants were more likely to have lower DBP in males (β = − 1.382, P = 0.017) and BP in females (β = − 1.776, P = 0.001 and β = − 1.156, P = 0.026 for SBP and DBP, respectively) compared to their unemployed counterparts. Only in male participants, a statistically significant association was observed between smoking and a decrease in SBP (β = − 1.496, P = 0.001). Maternal metabolic syndrome was associated with increased DBP in males (β = 0.843, P = 0.048) and increased SBP in females (β = 1.085, P = 0.035). Paternal metabolic syndrome was associated with higher DBP regardless of the participant’s sex (β = 1.238, P = 0.002 and β = 1.010, P = 0.029 for males and females, respectively. Furthermore, each unit increase in participants BMI was significantly associated with elevated SBP (β = 1.063 and β = 0.877 for males and females, respectively) and DBP (β = 0.453 and β = 0.452 for males and females, respectively) in both sexes.

**Table 3:** The association between LTPA trend and BP in male and female participants: the linear mixed model results.

	Males				Females			
	SBP		DBP		SBP		DBP	
	β	P-value	β	P-value	β	P-value	β	P-value
Intercept	76.746	** < 0.001**	50.516	** < 0.001**	79.795	** < 0.001**	54.648	** < 0.001**
Time	− 1.360	**0.006**	0.088	0.786	− 0.416	0.208	− 0.824	**0.014**
Age (Year)	0.425	**0.032**	0.426	**0.003**	0.027	0.775	0.199	**0.034**
Education								
Illiterate/Primary (ref)	0	–	0	–	0	–	0	–
Secondary	2.153	**0.004**	1.563	**0.006**	0.187	0.732	− 0.173	0.714
Higher	2.503	**0.020**	1.430	0.094	0.881	0.282	0.981	0.228
Marital status								
Married (ref)	0	–	0	–	0	–	0	–
Single	− 1.374	0.068	− 0.412	0.507	− 0.876	0.171	− 0.795	0.053
Occupation								
Unemployed (ref)	0	–	0	–	0	–	0	–
Employed	− 0.007	0.994	− 1.382	**0.017**	− 1.776	**0.001**	− 1.156	**0.026**
Smoking								
No (ref)	0	–	0	–	0	–	0	–
Yes	− 1.496	**0.001**	− 0.414	0.461	− 0.645	0.320	0.198	0.764
BMI (kg/m^2^)	1.063	** < 0.001**	0.453	** < 0.001**	0.877	** < 0.001**	0.452	** < 0.001**
Maternal Mets								
No (ref)	0	–	0	–	0	–	0	–
Yes	0.339	0.587	0.843	**0.048**	1.085	**0.035**	0.592	0.164
Paternal Mets								
No (ref)	0	–	0	–	0	–	0	–
Yes	1.166	0.088	1.238	**0.002**	0.875	0.126	1.010	**0.029**
LTPA (MET-hour/week)	− 0.004	0.595	− 0.009	0.269	0.006	0.729	0.027	0.111

The models were adjusted for age, height, marital status, education, and parental Mets.

β represents the mean BP changes in males and females due to each follow-up examination, each unit changes in LTPA, and influential factors status.

BMI, body mass index; SBP, systolic blood pressure; DBP, diastolic blood pressure; LTPA, leisure time physical activity.

Significant values are in [bold].

## Discussion

The present study investigated the association between LTPA and BP changes from adolescence to adulthood during more than a decade follow-up. Current results showed a sex-specific pattern in both LTPA and BP over time. While insufficient LTPA was a prevalent characteristic in both sexes, it exhibited a greater prevalence among females compared to males across all study measurements. Conversely, a more significant decline in LTPA was observed in males compared to their female counterparts in the long-term. Regarding BP, our results indicated a notable increasing trend exclusively among male participants. Moreover, after adjusting for potential individual and parental confounders, no significant association was observed between LTPA and BP changes over time in both sexes within the study population.

​In line with other studies in Iran^22,38^ and other countries^18,39^, most participants had insufficient LTPA. Although similar to other studies, adolescent males experience more decreasing changes in LTPA, this similarity was not found in the LTPA ratio among male and female participants over study assessments. We expected a smaller slope of decreasing changes in females' LTPA^40^, whereas the male-to-female LTPA ratio was nearly constant at about two in our follow-up examinations. One of the possible reasons might be the existing limitations and obstacles for females' PA in the studied society^41^.

Our results showed the sex-specific trend of SBP and DBP from adolescence to young adulthood. In line with other studies, the increasing trend of SBP in adolescent males will become constant at the beginning of adulthood, while in females, this stationary state for SBP was also visible from late adolescence^42–44^. The results of this study confirm the SBP changes reported for males and females after puberty toward early adulthood^45^. The similarity between the results of similar studies on SBP can not be generalized to DBP. However, In line with other studies, DBP in late adolescent males and females has continued the upward pattern until adulthood^42,44,46^; in some other studies, a steady or even decreasing DBP trend in late adolescence, especially in females, may be observed^43,47^. Regarding BMI, although the BMI of both sexes, similar to other study results had an upward trend in all follow-up periods^10^, the BMI effect on SBP and DBP is still unclear. Our study observed a significant rise in both SBP and DBP for both sexes in line with other research, as BMI increased in our study population^48^. However, some researchers argue for an opposite relationship^49^.

The present study showed no significant association between LTPA and BP changes over time in both sexes after adjusting potential confounders. There is no study aimed at investigating the relationship of LTPA and BP during the transitional period from adolescence to adulthood. Most studies conducted to investigate the effect of PA on BP changes during adolescence or adulthood revealed the common knowledge of the significant positive effect of LTPA on SBP and DBP^27,50,51^. However, it is important to highlight that upon reviewing other research, some other studies have produced inconclusive or differing results regarding the effect of PA on BP^14–17^. It is worth noting that these changes, whether positive or negative, in response to PA often occur within the normal range of BP in most of the studies. The observed changes might not be substantial enough to alter the clinical classification of BP. While these alterations are significant in the context of public health interventions, they tend to follow the “law of initial values,” meaning that individuals with higher initial BP values tend to experience more pronounced changes than those with initially normal BP^52^.

Within our study population, a significant proportion of participants in both sexes exhibited insufficient levels of LTPA. On average, the LTPA during adolescence consistently fell short of the recommended levels outlined by the WHO for both sexes, with more pronounced discrepancy in female participants. In adulthood, the situation was characterized by males having LTPA levels below the recommended minimum, while female adults engaged in LTPA that was merely half of the recommended amount. It is noteworthy that certain studies have suggested that engaging in 15–25 MET-hours/week of LTPA may reduce the risk of cardiovascular mortality^53^. Additionally, other research has proposed that maintaining approximately double the current minimum PA guideline levels at the beginning of young adulthood may help protect against the incidence of HTN^27^. Therefore, it is not unexpected to observe such an effect of LTPA on BP within our participants.

Based on the local and national data, there is a prevalent lack of sufficient LTPA among Iranian men and women in both urban and rural areas throughout their lifespan. In addition, healthy adolescents and young adults may not possess a comprehensive understanding of the potential risks associated with insufficient PA, which may potentially influence their levels of PA. This issue may rooted in existing controversial findings regarding the association between LTPA and BP changes over time. In this regard, certain studies have indicated that different levels of PA do not uniformly impact health-related variables, whether in adolescence or adulthood. These studies suggest that the increase in PA may not be directly proportional to improvements in health-related variables. In unadjusted models, some levels of PA have even been associated with increased SBP, DBP, BMI, and higher mortality rates related to conditions like stroke, coronary heart disease, or cardiovascular disease^24,54^. In other words, it appears that the effects of PA on BP can differ across populations with distinct characteristics. Therefore, additional research is needed, particularly within specific target populations, to develop a more precise understanding of the exact impact on individuals within these particular groups.

To the best of our knowledge, this is the first study using a linear mixed model to examine the association of LTPA and adolescents' SBP and DBP toward young adulthood regarding individual and intrapersonal differences over time. This model prevents false positive associations due to population or related structure, providing a more appropriate view of the participants’ variables. On the other hand, this study was implemented in the context of Tehran Lipid and Glucose Study (TLGS) as one of the oldest valid cohorts in the Middle East region. Nevertheless, the study has some limitations. Since this study is part of a community study and there is a possibility of missing some data during the data collection process, the imputation method has been used to compensate for the missing data. In addition, it was impossible to measure LTPA objectively, and a questionnaire was used for this purpose, which may increase recall bias and lead to underestimation of some relationships with risk factors in self-reporting methods compared to objective measurements. In addition, due to different age-specific criteria for BP classification in adolescence, the effect of LTPA on changing HTN grouping was not evaluated. Moreover, potential mediating and moderating factors in LTPA and BP association, including the participants’ emotional states, as well as environmental and socio-economic factors, have not been evaluated. Our study sample consisted of participants in Tehran, thus potentially limiting the generalizability of the findings to a broader population.

## Conclusion

The current longitudinal study showed no association between LTPA and changes in BP from adolescence to young adulthood. This lack of impact can largely be attributed to the low risk of developing high BP during the observed period, the prevalent lack of recommended levels of LTPA among the population, and a general lack of awareness regarding the detrimental impact of physical inactivity on cardiovascular health. These findings highlight the need for determining suitable levels of LTPA to reduce cardio-vascular risk among the Iranian population and planning tailored strategies aimed at promoting LTPA and increasing cardiovascular health literacy among the Iranian adolescents, with particular emphasis on females.

## Data Availability

The datasets used during the current study are available from the corresponding author on reasonable request.
